# Supplementary figures and images for: Lysinibacillus macroides 38352 isolated from traditional Chinese fermented foods: a dual effect on ochratoxin A detoxification and immune suppression alleviation
Source: Microbiol Spectr. 2026 Jan 21;14(3):e02363-25. doi: 10.1128/spectrum.02363-25 (PMC12955493; doi:10.1128/spectrum.02363-25)

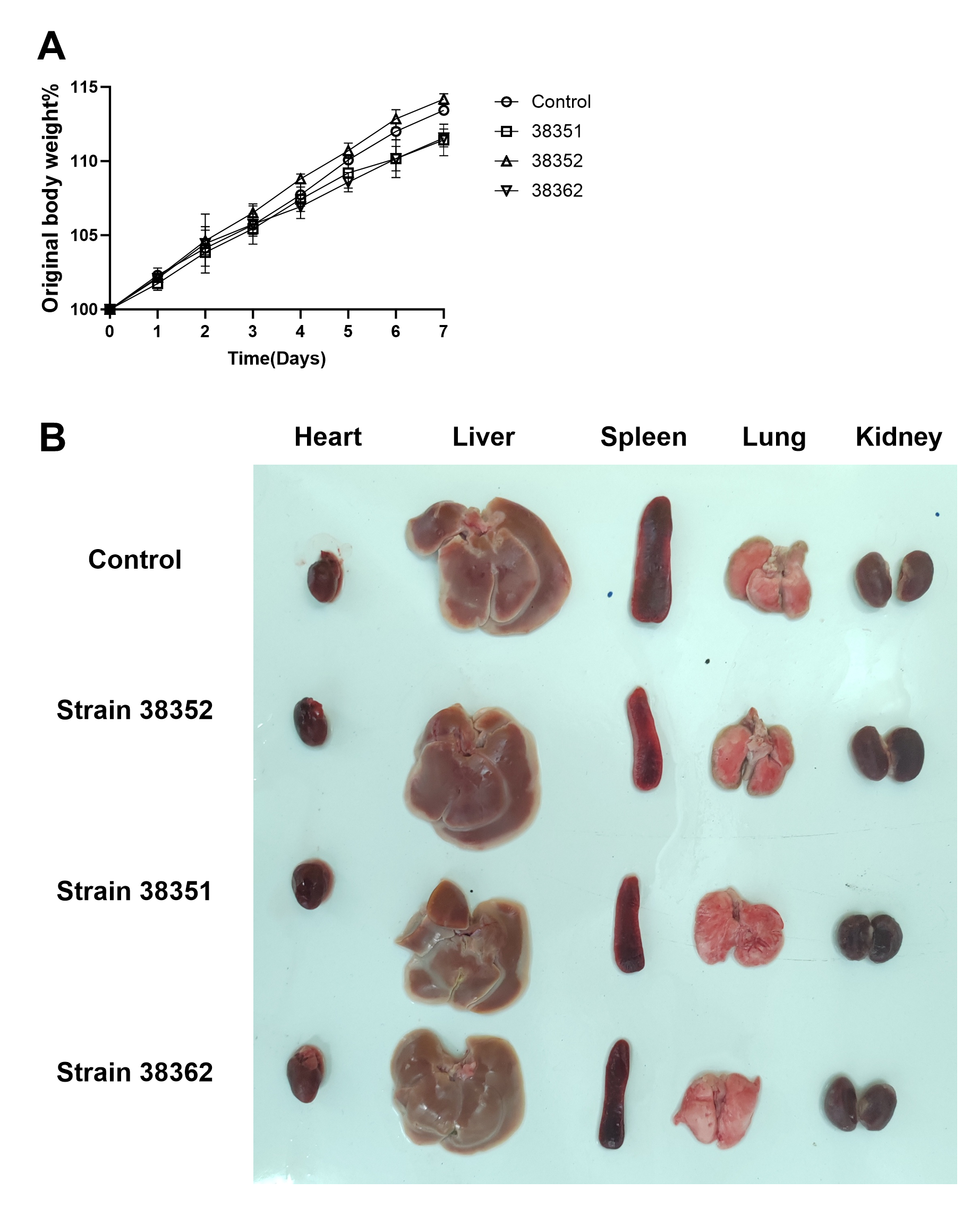

Supplement: Figure S1 — (A) Results of body weight change in mice; (B) results of mouse autopsy. [file spectrum.02363-25-s0001.tif]

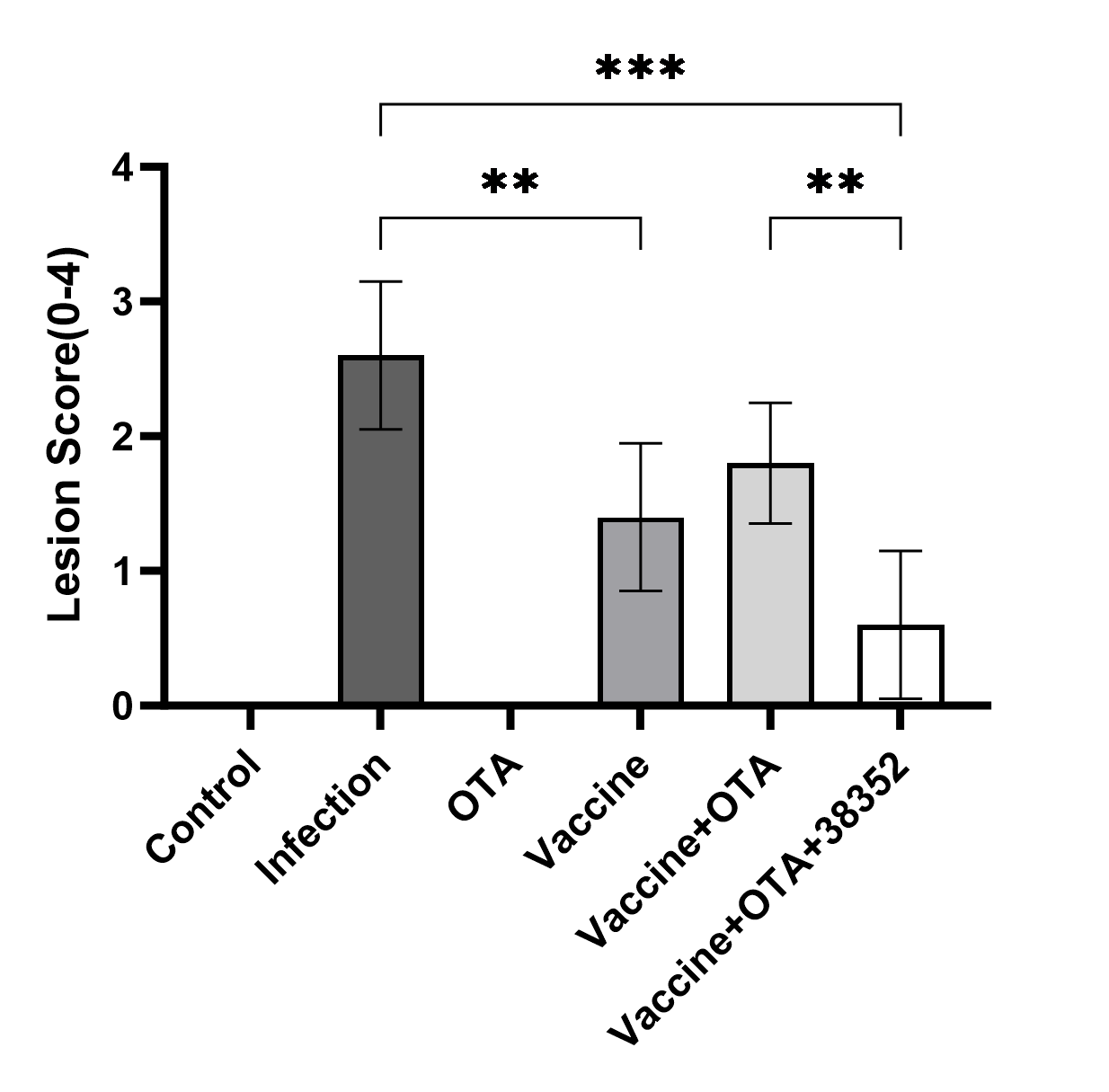

Supplement: Figure S2 — Lesion score results of each experimental group. [file spectrum.02363-25-s0002.tif]
