# Supplementary material for: Lysinibacillus macroides 38352 isolated from traditional Chinese fermented foods: a dual effect on ochratoxin A detoxification and immune suppression alleviation
Source: Microbiol Spectr. 2026 Jan 21;14(3):e02363-25. doi: 10.1128/spectrum.02363-25 (PMC12955493; doi:10.1128/spectrum.02363-25)
Supplement: Supplemental legends — Legends for supplemental material. [file spectrum.02363-25-s0003.docx]

supplemental legends

Figure S1. (A) Results of body weight change in mice. (B) Results of the mice autopsy.

Figure S2. Lesion score results of each experimental group.

Table S1. PCR primers sequences.

Table S2. Probiotic properties of isolates.

Table S3. Diameter of hemolytic ring in each experimental group.
