# Supplementary material for: Lysinibacillus macroides 38352 isolated from traditional Chinese fermented foods: a dual effect on ochratoxin A detoxification and immune suppression alleviation
Source: Microbiol Spectr. 2026 Jan 21;14(3):e02363-25. doi: 10.1128/spectrum.02363-25 (PMC12955493; doi:10.1128/spectrum.02363-25)
Supplement: Table S1 — PCR primer sequences. [file spectrum.02363-25-s0004.docx]

Table S1 PCR primers sequences

| gene | primer | sequence（5'–3'） |
| --- | --- | --- |
| β-actin | β-actin-F | GAGAAATTGTGCGTGACATCA |
|  | β-actin-R | CCTGAACCTCTCATTGCC |
| IL-1β | IL-1β-F | ACTGGGCATCAAGGGCTA |
|  | IL-1β-R | GGTAGAAGATGAAGCGGGTC |
| IL-4 | IL-4-F | GTGCCCACGCTGTGCTTAC |
|  | IL-4-R | AGGAAACCTCTCCCTGGATGTC |
| IL-17 | IL-17-F | CTCCGATCCCTTATTCTCCTC |
|  | IL-17-R | AAGCGGTTGTGGTCCTCAT |
| IL-12 | IL-12-F | AGACTCCAATGGGCAAATGA |
|  | IL-12-R | CTCTTCGGCAAATGGACAGT |
| IFN-γ | IFN-γ-F | TAACTCAAGTGGCATAGATGTGGAAG |
|  | IFN-γ-R | GACGCTTATGTTGTTGCTGATGG |
