# Supplementary material for: Lysinibacillus macroides 38352 isolated from traditional Chinese fermented foods: a dual effect on ochratoxin A detoxification and immune suppression alleviation
Source: Microbiol Spectr. 2026 Jan 21;14(3):e02363-25. doi: 10.1128/spectrum.02363-25 (PMC12955493; doi:10.1128/spectrum.02363-25)
Supplement: Table S2 — Probiotic properties of isolates. [file spectrum.02363-25-s0005.docx]

Table S2 Probiotic properties of isolates

|  | **Hemolytic** | **pH tolerance** | **pH tolerance** | **pH tolerance** | **Bile** |
| --- | --- | --- | --- | --- | --- |
|  | activity | (pH 1) | (pH 2) | (pH 12) | tolerance |
| 38351 | – | +++ | +++ | +++ | +++ |
| 38352 | – | +++ | +++ | +++ | +++ |
| 38362 | – | +++ | +++ | +++ | +++ |
| +++ > 90% survival rate; ++ > 80% survival rate; + > 70% survival rate; –, nil | | | | | |
