# Supplementary material for: Lysinibacillus macroides 38352 isolated from traditional Chinese fermented foods: a dual effect on ochratoxin A detoxification and immune suppression alleviation
Source: Microbiol Spectr. 2026 Jan 21;14(3):e02363-25. doi: 10.1128/spectrum.02363-25 (PMC12955493; doi:10.1128/spectrum.02363-25)
Supplement: Table S3 — Diameter of hemolytic ring in each experimental group. [file spectrum.02363-25-s0006.docx]

Table S3. Diameter of hemolytic ring in each experimental group

| Group | Control | OTA | Vaccine | Vaccine + OTA | Vaccine + OTA + 38352 |
| --- | --- | --- | --- | --- | --- |
| Diameter(mm) | 16 | 16.3 | 15 | 15.5 | 13.5 |
